# Advances in nanotechnology-enabled drug delivery for combining PARP inhibitors and immunotherapy in advanced ovarian cancer

**DOI:** 10.17305/bb.2023.9757

**Published:** 2024-04-01

**Authors:** Lama Abujamous, Abderrezzaq Soltani, Hamda Al-Thawadi, Abdelali Agouni

**Affiliations:** 1 Department of Basic Medical Sciences, College of Medicine, QU Health, Qatar University, Doha, Qatar; 2 Office of Vice President for Research and Graduate Studies, Qatar University, Doha, Qatar; 3 Office of Vice President for Medical and Health Sciences, Qatar University, Doha, Qatar; 4 Department of Clinical Pharmacy and Practice, College of Pharmacy, QU Health, Qatar University, Doha, Qatar; 5 Department of Pharmaceutical Sciences, College of Pharmacy, QU Health, Qatar University, Doha, Qatar

**Keywords:** Ovarian carcinomas, combination therapy, poly (ADP-ribose) polymerase (PARP) inhibitors (PARPis), immune checkpoint inhibitors (ICIs), nanomedicine

## Abstract

Advanced ovarian cancer is a malignancy that spreads beyond the ovaries to the pelvis, abdomen, lungs, or lymph nodes. Effective treatment options are available to improve survival rates in patients with advanced ovarian cancer. These include radiation, surgery, chemotherapy, immunotherapy, and targeted therapy. Drug resistance, however, remains a significant challenge in pharmacotherapeutic interventions, leading to reduced efficacy and unfavorable patient outcomes. Combination therapy, which involves using multiple drugs with different mechanisms of action at their optimal dose, is a promising approach to circumvent this challenge and it involves using multiple drugs with different mechanisms of action at their optimal dose. In recent years, nanotechnology has emerged as a valuable alternative for enhancing drug delivery precision and minimizing toxicity. Nanoparticles can deliver drugs to specific cancer cells, resulting in higher drug concentrations at the tumor site, and reducing overall drug toxicity. Nanotechnology-based drug delivery systems have the potential to improve the therapeutic effects of anti-cancer drugs, reduce drug resistance, and improve outcomes for patients with advanced ovarian cancer. This literature review aims to examine the current understanding of combining poly (ADP-ribose) polymerase (PARP) inhibitors and immunotherapy in treating advanced ovarian cancer and the potential impact of nanotechnology on drug delivery.

## Introduction

Ovarian cancer is a leading cause of mortality among women globally [1]. It is often referred to as a “silent killer” due to the nonspecific symptoms in its early stages [2]. Consequently, symptoms signaling a severe condition may not be recognized until the disease has progressed to an advanced stage. Typical symptoms include pelvic pain, bloating, stomach discomfort, and urinary problems [3]. The high mortality rates of ovarian cancer are indeed attributed to its late diagnosis, with 60% of women being diagnosed in advanced stages [4]. Treatment options include debulking surgery or cytoreduction, chemotherapy, and radiation therapy, while advanced therapeutic approaches, such as targeted therapy, immunotherapy, and hormone therapy are also being considered [1].

Clinical researchers are continually exploring novel strategies and effective trial designs to test anticancer drugs. Many of these novel strategies are underpinned by the shift toward molecularly targeted drug development. Creating effective clinical trials is more challenging than ever due to the constrained resources and the niche patient groups for whom precision-targeted drugs are appropriate [5]. The past decade has seen exponential advances in ovarian cancer treatment, with numerous new investigational combination drugs and drug approvals. Combinations designed to overcome resistance mechanisms have garnered special attention [6]. Determining the best treatment regimens, evaluating new agents effectively, and improving patient outcomes remain elusive.

The development of nanomaterials has paved the way for a new frontier in cancer treatment with promising research in drug delivery systems [7]. Nanoparticles could potentially offer superior antineoplastic benefits compared to standard anticancer drugs, due to ease of manufacture, high efficacy, low toxicity, and aggressive tumor-targeting capabilities. These drug delivery systems include delivery carriers of anticancer drugs and target molecules [8].

This review focuses on two main areas: the combined and repurposed use of poly (ADP-ribose) polymerase (PARP) inhibitors (PARPis) and immune checkpoint inhibitors (ICIs) in ovarian cancer, as well as the potential of nanomedicine as a novel approach to drug delivery. While existing literature addresses targeted therapy for ovarian cancer, there is no study that provides an in-depth analysis of personalized drug delivery methods specific to ovarian cancer.

## Human ovarian cancer

Ovarian cancer is a prevalent malignancy with a high mortality rate, ranking as the world’s seventh most common malignant tumor and one of the most prevalent gynecologic cancer types [9]. Its mortality rate stands third behind cervical and uterine cancers [10]. Postmenopausal women, especially those over 65 years old, are at a higher risk [11]. The incidence of ovarian cancer has been rising with the mortality rate surging by 20.1% annually per 100,000 individuals in the Middle East and North Africa (MENA) region since 1990 [12]. Within the Gulf region, Bahrain has the highest age-standardized rate (ASR) per 100,000 women, followed by Qatar, Kuwait, Oman, the United Arab Emirates, and Saudi Arabia [13]. The incidence rate of ovarian cancer is highest in non-Hispanic white women, followed by Hispanic women, non-Hispanic black women, and Asian/Pacific Islander women [14].

The pathogenesis of ovarian cancer is linked to various risk factors, including regional epidemiological variations [10], genetic predispositions like hereditary breast and ovarian cancer (HBOC) syndrome, Lynch syndrome [15], advanced age at menopause, and hormone replacement therapy treatment [10]. Reproductive and hormonal factors, such as parity, contraceptive use, and breastfeeding, may reduce the risk of ovarian cancer. The relationship between risk factors and histotype varies due to etiological variations. For instance, patients with endometriosis are at a higher risk of developing mucinous ovarian cancer and clear cell ovarian cancer. Conversely, patients who undergo tubal ligation are at a reduced risk [16]. The association with other gynecological diseases and procedures, such as hysterectomy, pelvic inflammatory disease, polycystic ovarian syndrome, and ovarian cancer risk is less well defined [16]. Environmental and lifestyle factors, such as asbestos and talc powder exposure and cigarette smoking are also potential risk factors. What is certain is that further research is required to improve our understanding of the disease etiology and develop more effective prevention and early detection strategies [16].

The pathophysiology of ovarian cancer is complex, impacting different cell types in the ovaries, including non-epithelial cells, such as germ cells and specialized gonadal stromal cells, as well as epithelial cells [17]. Over 90% of diagnosed cases of ovarian cancer are carcinomas, which are ovarian tumors with epithelial differentiation, accounting for the majority of ovarian cancer-related deaths [17].

Pathologists divide epithelial ovarian carcinomas (EOCs) into five main types based on their microscopic appearance: high-grade serous carcinomas (HGSCs), endometrioid carcinoma (EC), clear cell carcinoma (CCC), low-grade serous carcinomas (LGSCs), and mucinous carcinoma (MC) [15, 17]. These different subtypes of EOCs have distinct clinical and molecular characteristics that impact the diagnosis, prognosis, and treatment strategy. HGSC is the most common and aggressive EOC subtype, typically chemoresistant, and commonly affects postmenopausal women [18].

Ovarian carcinomas can also be classified into two tumor types, I and II, based on their distinct characteristics [17]. Type I tumors are low-grade, non-aggressive, and genetically stable, often arising from benign tumors or endometriosis [19]. Common somatic mutations in Type I tumors impair cell signaling pathways or chromatin folding complexes, including B-Raf Proto-Oncogene (*BRAF*), Kirsten ras oncogene homolog (*KRAS*), or phosphatase and tensin homolog (*PTEN*) mutations found in ECs, CCCs, MCs, and LGSCs. Conversely, type II tumors are high-grade, physiologically aggressive, and prone to spreading from small lesions. HGSCs, the most common type II tumors, feature tumor protein 53 (*TP53*) mutations and multiple gene modifications (e.g., breast cancer gene or *BRCA1/2*), which lead to homologous recombination (HR) anomalies [15, 18, 20].

To evaluate ovarian cancer specimens, pathologists must report the histology, tumor grade, particularly for serous malignancies, and tumor stage, which refers to the degree of systemic involvement in other tissues/organs, according to the College of American Pathologists standard [21]. Prognostic predictions in ovarian cancer are challenging due to the diverse range of tumors. The standard treatment for advanced ovarian cancer involves primary cytoreductive surgery, followed by platinum-based chemotherapy. The aim of cytoreductive surgery is to establish an accurate diagnosis, remove disease-bearing tissue with poor perfusion, and reduce tumor size to improve the effectiveness of adjuvant treatment. Combination treatment with surgery and chemotherapy is the conventional approach to manage ovarian cancer [22, 23].

## Current treatment of ovarian cancer

The first-line treatment of ovarian carcinoma involves optimal debulking surgery followed by platinum/gemcitabine and taxane-based chemotherapy [24]. Despite the high response rate, recurrence is common within three years. In platinum-resistant cases (with relapse occurring within six months of treatment) or in platinum-refractory disease (progressing within four weeks following platinum-based chemotherapy), approximately 20%–25% of patients have a poor prognosis when they first relapse [25]. Single non-platinum agents like paclitaxel and pegylated liposomal doxorubicin (PLD) are often prescribed in platinum-resistant diseases with limited efficacy and poor outcomes. Given the diverse nature of clinical presentations of ovarian carcinomas, both at diagnosis and upon recurrence, it is imperative to devise better approaches to predict both immediate and long-term treatment outcomes for both newly diagnosed and recurrent cases [23].

Given the considerable risk of drug resistance following initial therapy with first-line treatment, it is paramount to consider a broader array of treatment strategies for upfront and recurrent settings to improve patient outcomes [23]. Targeted therapy is a promising approach to address tumor cell alterations that promote their growth, proliferation, and metastasis. Potential therapeutic targets in gynecological malignancies include tumor-intrinsic signaling pathways, angiogenesis, HR deficiency (HRD), hormone receptors, and immunologic factors [26]. Targeted therapy includes signaling pathway inhibitors, selective estrogen receptor down-regulators, anti-angiogenic agents, PARPis, and ICIs.

The U.S. Food and Drug Administration (FDA) has recently approved bevacizumab, pembrolizumab, and olaparib to treat recurrent metastatic or high-risk gynecological malignancies in selected patients [24]. Clinical applications of targeted drugs and other potential treatments are still being extensively investigated to develop better and more personalized treatment strategies for patients with ovarian carcinomas.

There are numerous factors that impact the selection of therapy for ovarian cancer. These include treatment indications, FDA approval, sensitivity to platinum-based therapies, outcomes of molecular studies, and testing strategies. Other important considerations include risk stratification, assessing the patient’s likelihood to respond to PARPis, and evaluating the efficacy of past treatments. Patient-specific factors like performance status, end-organ function, previous treatment side effects, toxicity profiles, patient adherence, and treatment costs should also be considered for a more holistic and patient-centered approach [27].

Surgery and chemoradiotherapy remain the gold standard therapy for ovarian cancer. The severe side effects associated with chemotherapy and radiation however underscore the need to explore alternative treatment strategies. Our enhanced understanding of ovarian cancer biology has paved the way for molecular targeted therapies which could potentially offer better patient outcomes. The pharmacotherapies currently available for the treatment of ovarian cancer and their drawbacks are summarized in Table 1.

**Table 1:** Currently available therapeutic options for ovarian cancer treatment

**Drug**	**Class**	**Target**	**Major drawbacks**	**Database**
Thiotepa	Organic thiophosphoric acids	DNA	Nausea, vomiting, loss of appetite, stomach pain, unusual tiredness or weakness, dizziness, headache, blurry vision, sore or red eyes, hair loss, risk for other cancers	NCI, Drug bank, MedlinePlus
Paclitaxel	Taxanes	Tubulin beta-1 chain, Apoptosis regulator Bcl-2, Microtubules	Myelosuppression, peripheral neuropathy, alopecia, hypersensitivity reactions	NCI, UTD, Drug bank
Doxorubicin	Anthracyclines	DNA, DNA topisomerase 2-alpha/2-beta	Risk of secondary malignancies, premature menopause	NCI, UTD, Drug bank
Topotecan	Camptothecin	DNA, DNA topoisomerase 1	Bone marrow suppression	NCI, UTD, Drug bank
Nivolumab	Immune checkpoint inhibitor	PD-1	Toxicity, including pneumonitis, colitis, hepatitis, endocrinopathies, nephritis, and skin reactions – May cause immune-mediated complications	UTD, Drug bank, **Not included in the FDA-approved list by NCI** **for ovarian cancer**
Pembrolizumab	Immune checkpoint inhibitor	PD-1	Toxicity, including pneumonitis, colitis, hepatitis, endocrinopathies, nephritis, and skin reactions – May cause immune-mediated complications	Drug bank, **Not included in the FDA-approved list by NCI for ovarian cancer**
Atezolizumab	Immune checkpoint inhibitor	PD-L1	Toxicity, including pneumonitis, colitis, hepatitis, endocrinopathies, nephritis, and skin reactions – May cause immune-mediated complications	Drug bank, **Not included in the FDA-approved list by NCI for ovarian cancer**
Mirvetuximab soravtansine	Monoclonal antibody (mAb)	Folate receptor alpha	Ocular toxicity, pneumonitis, peripheral neuropathy	NCI, UTD, Drug bank
Cyclophosphamide	Nitrogen mustard	DNA	Neutropenia, febrile neutropenia, fever, alopecia, nausea, vomiting, diarrhea.	NCI, UTD, Drug bank
Olaparib	PARP inhibitor	PARP1, PARP2, PARP3	MDS/AML, cardiovascular toxicity	NCI, UTD, Drug bank
Niraparib	PARP inhibitor	PARP1, PARP2, PARP3	MDS/AML, thrombocytopenia, anemia, neutropenia	NCI, UTD, Drug bank
Rucaparib	PARP inhibitor	PARP1, PARP2, PARP3	MDS/AML, gastrointestinal toxicity	NCI, UTD, Drug bank
Melphalan	Phenylalanine	DNA	Vomiting, ulceration of the mouth, diarrhea, hemorrhage of the gastrointestinal tract	NCI, Drug bank
Carboplatin	Platinum compounds	DNA	Neutropenia, hepatotoxicity	NCI, UTD, Drug bank
Cisplatin	Platinum compounds	DNA	Nephrotoxicity, hearing problems	NCI, UTD, Drug bank, MedlinePlus
Gemcitabine	Pyrimidine 2’-deoxyribonucleosides	DNA, Ribonucleoside-diphosphate reductase	Myelosuppression, paresthesia, severe rash	NCI, UTD, Drug bank
Docetaxel	Taxane	Microtubules	Myelosuppression, peripheral neuropathy, alopecia, hypersensitivity reactions	UTD, Drug bank, **Not included in the FDA approved list by NCI for ovarian cancer**
Pazopanib	VEGF inhibitor	VEGFR-1, VEGFR-2, VEGFR-3	Cardiovascular toxicity, hepatic toxicity, hypertension, gastrointestinal toxicity	UTD, Drug bank, **Not included in the FDA-approved list by NCI for ovarian cancer**
Bevacizumab	VEGF inhibitor	VEGF-A	Cardiovascular toxicity, gastrointestinal perforation, arterial thromboembolic events, hypertension	NCI, UTD, Drug bank

NCI: National Cancer Institute; UTD: Up-to-Date (an evidence-based medicine website); VEGF: Vascular endothelial growth factor; VEGFR: VEGF receptor; PD-1: Programmed death protein 1; FDA: Food and Drug Administration; PD-L1: Programmed death-ligand 1; PARP: Poly (ADP-ribose) polymerase; MDS/AML: Myelodysplastic syndrome/acute myeloid leukemia.

### PARP inhibitors (PARPis) in the treatment of ovarian cancer: Current status and future directions

In recent years, there has been a marked increase in research focus on improving the efficacy of existing treatments for advanced ovarian cancer. While platinum-based chemotherapy remains the treatment mainstay for advanced ovarian cancer, most patients develop resistance and experience relapse within the first two years of treatment [28]. This pressing challenge justifies the quest of the research community to explore and develop innovative approaches to manage advanced ovarian cancer.

One of the most promising treatment strategies for advanced ovarian cancer involves the use of PARPis. These inhibitors target DNA repair and are primarily active in cells with defective DNA repair via the HR pathway. HRD is evident in cells with mutant germline *BRCA* function and in a high percentage of non-*BRCA*-mutated ovarian cancers [29]. *BRCA* mutations are found in nearly 15% of all EOCs and up to 22.6% of HGSOCs, with somatic *BRCA* mutations identified in approximately 7% of high-grade serous EOCs [30]. PARPis have demonstrated efficacy beyond germline *BRCA* mutations in DDR deficiency. Other somatic mutations in genes, such as Ataxia telangiectasia and Rad3-related protein (*ATR*), Checkpoint kinase 1/2 (*CHK1/2*), BRCA1-associated RING domain protein 1 (*BARD1*), BRCA1-interacting protein 1 (*BRIP1*), Deleted in split hand/split foot 1 (*DSS1*), Nijmegen breakage syndrome 1 mutated gene (*NBS1*), Partner and Localizer of Breast Cancer 2 (*PALB2*), RAD51 recombinase (*RAD51*), cyclin-dependent kinase 12 (*CDK12*), Ataxia-Telangiesctasia Mutated (*ATM*), Fanconi anemia complementation group, Partner and Localizer of BRCA2 (*PALB2*), X-Ray Repair Cross Complementing 2 (*XRCC2*), X-ray repair cross-complementing group 3 (*XRCC3*), *TP53*, or *PTEN* may serve as diagnostic biomarkers for PARPis treatment in various cancers without *BRCA* mutations [6].

Moreover, PARPis result in synthetic lethality, which is the simultaneous perturbation of two genes that leads to cellular or organismal death [31]. Synthetic lethality may be one of the most promising pathways in the realm of personalized medicine which exploits dysregulated DNA damage. PARP1 undergoes poly (ADP) ribosylation because of its affinity to DNA strand breaks. The process initiates the recruitment of additional DNA repair pathways as well as poly (ADP) ribosylation [32]. When PARP1 is inhibited, single-strand breaks (SSB) lesion repair fails, while double-strand breaks (DSB) repair remains unaffected. Because PARP1 enzymatic activity is reduced in *BRCA1/2*-deficient cells, SSBs are transformed into DSBs and are repaired by HR, resulting in cell cycle arrest and death [6].

PARPis have been shown to improve progression-free survival and overall survival in patients with platinum-sensitive recurrent ovarian cancer and subsequent high-grade ovarian cancer which did not progress following first-line platinum-based chemotherapy [6]. Anti-angiogenic therapy and PARPis have recently been added to standard treatment protocols. Several studies have demonstrated that such combinations improve progression-free survival and overall survival in women with ovarian cancer [33]. However, further research is needed to determine the most suitable patient candidates who will benefit the most from these treatment approaches. Notably, the impact of PARPis on immune cells may amplify the anticancer activity of ICIs, as dying tumor cells can trigger an immune response that is mediated in part by various transcriptional factors and chemokines [1, 7]. Anticancer drugs inhibiting the immune checkpoint could be boosted if the immune system responds to dying tumor cells [34]. One crucial aspect that is still under investigation is to discern which patients benefit the most from combination therapy with PARPis and ICIs [1]. In summary, PARPis offer a promising therapeutic approach for patients with advanced ovarian cancer, especially those with HRD. Combination therapy with ICIs could prove to be a viable treatment option to further improve patient outcomes.

**Figure 1.**
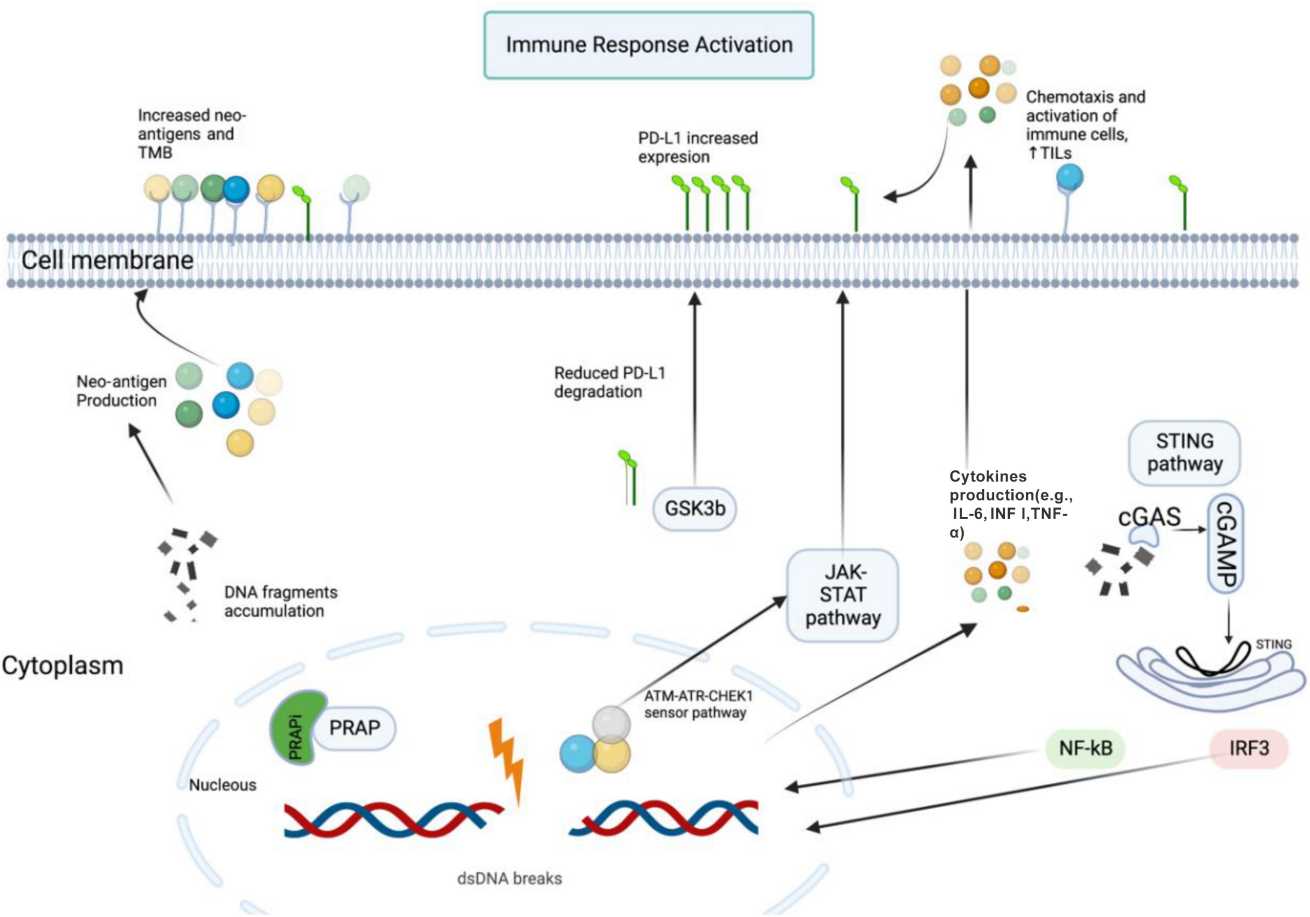
**Interaction between PARPis and ICIs.** When double-strand DNA breaks (DSBs) occur, DNA fragments accumulate in the cytoplasm, and the PARP complex is blocked by PARPis. This results in the collection of neo-antigens on the cell surface, which are recognized by antigen-presenting cells (APCs), triggering an immunological response. The cytosolic sensor cGMP-AMP synthetase (cGAS) identifies fragments of DNA, activating cyclic GMP-AMP (cGAMP), which then activates the STING pathway. The STING pathway modulates transcription factors like nuclear factor-κB (NF-κB) and interferon regulatory factor 3 (IRF3), leading to the transcription of cytokines, including interferon (IFN), interleukin 6 (IL-6), and tumor necrosis factor-α (TNF-α). IFN increases the expression levels of programmed cell death ligand 1 (PD-L1). DSBs activate the ataxia-telangiectasia mutated (*ATM*)-(*ATR*) serine/threonine kinase (*ATR*)-checkpoint kinase 1 (*CHEK1*) and signal transducers and activators of transcription (STAT)-IRF sensor pathways, leading to enhanced PD-L1 expression. DSBs also inhibit glycogen synthase kinase 3β (GSK-3β), which is involved in PD-L1 proteasomal degradation, resulting in increased PD-L1 cellular expression. PARPis: Poly (ADP-ribose) polymerase (PARP) inhibitors; ICIs: Immune checkpoint inhibitors; PARP: Poly (ADP-ribose) polymerase; TIL: Tumor-infiltrating lymphocytes; TMB: Tumor mutation burden.

### Immunotherapy with immune checkpoint inhibitors (ICIs) in ovarian cancer

Immunotherapy has revolutionized cancer therapy with ICIs playing a central and promising role in combating various malignancies. These anticancer agents are designed to block mechanisms that inhibit the immune response [34] by targeting programmed death protein 1 (PD-1)/programmed death-ligand 1 (PD-L1), or cytotoxic T-lymphocyte-associated protein 4 (CTLA-4) [35]. Among gynecologic cancers, endometrial cancer is most responsive to ICIs, while ovarian, cervical, and vulvar cancer are significantly less so [36]. Combination therapy of ICIs and other anticancer agents, including PARPis, has shown promise in treating ovarian, cervical, and vulvar cancer as reflected in findings of several phases I and II clinical trials [37]. ICIs, whether administered alone or in combination with other therapies, have been shown to prolong the survival of patients with advanced diseases, such as melanoma, genitourinary, lung, and gastric cancer. However, the efficacy of ICIs varies depending on the cancer type and even within the same tumor tissue cohort. Notably, the benefits of ICIs therapy may be limited in some common tumor types, such as prostate and breast cancer [37].

In conclusion, the long-term use of ICIs as a single therapy is marked with limited efficacy. Combination therapies, particularly with PARPis, may help in overcoming resistance and augmenting the beneficial effects of ICIs [38].

### Combining PARPis and ICIs in ovarian cancer treatment

PARPis or ICIs monotherapy often leads to resistance, necessitating combination therapies to bypass resistance and enhance efficacy [37]. PARPis affect the tumor immune microenvironment through molecular and cellular mechanisms, making them a compelling candidate for combination with ICIs. PARPis have been linked to increased PD-L1 expression, potentially mediated by the inactivation of glycogen synthase kinase (GSK)-3β, leading to decreased PD-L1 degradation by proteasomes and increased surface expression [38, 39]. Additionally, PARPis activate the STING pathway to promote PD-L1 expression in response to interferon release and upregulate PD-L1 through the ATM-ATR-CHEK1 pathway [38] (Figure 1).

Research has shown that cancer cells increase the production of PD-L1 in response to DNA damage caused by PARPis, which inhibits T-cell response and may reduce treatment efficacy [6, 37]. This suggests a potential role for PARPis in treating platinum-resistant cancers by counteracting the effects of PD-L1. Furthermore, the immunomodulatory effect of PARPis justifies their combination with ICIs, as this synergy has shown great promise in advanced ovarian cancer treatment [37, 38].

To date, four clinical trials (TOPACIO/Keynote162/NCT02657889, NCT02484404, MEDIOLA/NCT02734004, and NCT03574779) investigating the efficacy of combining PARPis with ICIs have provided insights into the potential benefits of the combination approach been published, including [38]. Unlike BRCA inhibitors monotherapy, which proved more effective in patients with *BRCA* mutations, these studies showed there was no significant difference in response or survival rates based on BRCA or HR status in the combination therapy [37]. This suggests that the importance of *BRCA* mutations may be diminished in the context of PARP and ICI combination therapy, paving the possibility open for further investigation in future trials [37, 38].

In conclusion, the combination therapy improves anti-tumor immune response and boosts the efficacy of PARPis in ovarian cancer. Clinical trials in women with ovarian cancer have shown promising results by displaying higher response rates and progression-free survival.

### Challenges in drug delivery for combining PARPis and ICIs

Advances in our understanding of cancer microenvironments and intracellular signaling pathways have paved the way to develop targeted molecular therapies for ovarian cancer. These therapies can attack tumor cells or their microenvironment by targeting molecules that induce cell death or promote cell survival [5]. Traditional chemotherapy using cytotoxic drugs, targeted molecular inhibitors, and immunotherapy can fail when ovarian cancer cells acquire resistance, metastasize, or when patients no longer tolerate the treatment due to severe side effects.

To surmount these challenges, targeted delivery systems like antibody-drug conjugates (ADCs), peptide/folate/aptamer-drug conjugates, polymer-drug conjugates, ligand-functionalized nanomedicines, and dual-targeted nanomedicines have come to the fore over the past decade [24]. These delivery systems have the potential to improve the efficacy of ovarian cancer chemotherapy and molecular therapy in both preclinical and clinical settings by enhancing therapeutic agent selectivity, overcoming drug resistance, and minimizing systemic toxicity.

Specifically, nanoconjugates, branching dendrimers, liposomes, nanostructured lipid formulations, and polymer nanomicelles, in particular, are nanocarriers that have been developed extensively to deliver drugs to cancer cells [40]. These biopharmaceutical delivery systems offer several advantages, including enhanced drug biodegradability, increased therapeutic impact, biocompatibility, non-toxic and non-inflammatory properties, and potential for scalable manufacturing [41]. In the context of chemotherapeutic systems, it is crucial that nanoformulations have a high drug-loading capacity, the ability to dissolve pharmaceuticals in the inner core, and the capability to preferentially accumulate in tumor tissue via passive or active targeting mechanisms [13].

## Conclusion and future perspectives

Treating ovarian cancer remains a significant clinical challenge due to the persistent issue of chemoresistance and the lack of additional treatment options to overcome it effectively. The biological mechanisms underlying drug resistance remain poorly understood. Identifying genes associated with chemotherapy response and patient survival may provide valuable insights to guide prognosis and treatment decisions and prevent chemoresistance. Unlike most malignancies, ovarian cancer has limited therapy options, making it paramount to gain a deeper understanding of the molecular pathways involved in drug resistance to develop targeted interventions that combat or eliminate resistance.

In a recent study by Giannini et al., significant concerns pertaining to the utilization of PARPis have been thoughtfully addressed. A notable concern highlighted is the reoccurrence of the disease in a significant number of individuals who received treatment with these inhibitors within a 30-month span after their first diagnosis [42]. There are lingering uncertainties surrounding the management of relapses, the optimal positioning of PARPis as either first-line or second-line therapies, and whether patients can be managed effectively with PARPis alone. To address these concerns, current clinical studies are actively exploring diverse strategies, such as the integration of PARPis with chemotherapy, antiangiogenic therapy, ICIs, or other therapeutic interventions that modulate the DNA damage response. It is noteworthy that the initial results derived from these trials indicate that the combination of PARPis with chemotherapy may not yield substantial improvements in patient outcomes. On the other hand, the potential of PARPis in conjunction with antiangiogenic therapy is encouraging, as is the prospect of utilizing PARPis to augment the effectiveness of ICIs. In addition, scholars are currently engaged in the investigation of methodologies aimed at addressing resistance to PARPis by targeting the cell cycle [42].

Advances in technology and analytical tools have provided researchers with a more nuanced understanding of ovarian tumors. They are now able to identify specific biomarkers crucial for predicting tumor response to targeted therapy. Overcoming drug resistance is indeed a significant challenge that could potentially be overcome by promising combination treatments that inhibit multiple targets but this approach warrants further investigation. Moreover, targeted drug delivery via nanoparticles has shown significant therapeutic potential to improve cancer treatment efficacy compared to traditional chemotherapy. Nanoparticules-based drug delivery systems, such as nanocarriers, hold immense promise as a primary treatment option for ovarian cancer and could also play a pivotal role in managing other types of cancer in the future.
